# H_2_O_2_ drives the transition from conchocelis to conchosporangia in the red alga *Pyropia haitanensis* with promotion facilitated by 1-Aminocyclopropane-1-carboxylic acid

**DOI:** 10.3389/fpls.2024.1379428

**Published:** 2024-03-12

**Authors:** Tingting Niu, Haike Qian, Haimin Chen, Qijun Luo, Juanjuan Chen, Rui Yang, Peng Zhang, Tiegan Wang

**Affiliations:** ^1^ State Key Laboratory for Managing Biotic and Chemical Threats to the Quality and Safety of Agro-products, Ningbo University, Ningbo, Zhejiang, China; ^2^ Collaborative Innovation Center for Zhejiang Marine High-efficiency and Healthy Aquaculture, Ningbo University, Ningbo, Zhejiang, China; ^3^ Department of Genetic breeding, Zhejiang Mariculture Research Institute, Wenzhou, China

**Keywords:** *Pyropia haitanensis*, conchosporangia, H_2_O_2_, 1-Aminocyclopropane-1-carboxylic acid, life cycle

## Abstract

The Bangiales represent an ancient lineage within red algae that are characterized by a life history featuring a special transitional stage from diploid to haploid known as the conchosporangia stage. However, the regulatory mechanisms governing the initiation of this stage by changes in environmental conditions are not well understood. This study analyzed the changes in phytohormones and H_2_O_2_ content during the development of conchosporangia. It also compared the gene expression changes in the early development of conchosporangia through transcriptome analysis. The findings revealed that H_2_O_2_ was shown to be the key signal initiating the transition from conchocelis to conchosporangia in *Pyropia haitanensis*. Phytohormone analysis showed a significant increase in 1-aminocylopropane-1-carboxylic acid (ACC) levels during conchosporangia maturation, while changes in environmental conditions were found to promote the rapid release of H_2_O_2_. H_2_O_2_ induction led to conchosporangia development, and ACC enhanced both H_2_O_2_ production and conchosporangia development. This promotive effect was inhibited by the NADPH oxidase inhibitor diphenylene iodonium and the H_2_O_2_ scavenger N, N’-dimethylthiourea. The balance of oxidative–antioxidative mechanisms was maintained by regulating the activities and transcriptional levels of enzymes involved in H_2_O_2_ production and scavenging. Transcriptome analysis in conjunction with evaluation of enzyme and transcription level changes revealed upregulation of protein and sugar synthesis along with modulation of energy supply under the conditions that induced maturation, and exogenous ACC was found to enhance the entire process. Overall, this study demonstrates that ACC enhances H_2_O_2_ promotion of the life cycle switch responsible for the transition from a vegetative conchocelis to a meiosis-preceding conchosporangia stage in Bangiales species.

## Introduction

Life history patterns can be construed as adaptive strategies with features that have evolved in response to diverse selective pressures. Plants and seaweeds exhibit control over their growth and reproduction, adjusting in response to environmental changes to optimize their life-cycle progression and better adapt to the variable natural conditions they encounter (Suda and Mikami, 2020). Unraveling the regulatory mechanisms governing these life-cycle strategies is crucial to understanding how seaweeds integrate environmental signals and tailor their life cycle accordingly, as well as to augmenting production in mariculture farming of economically significant marine resources.

**Figure 1:**
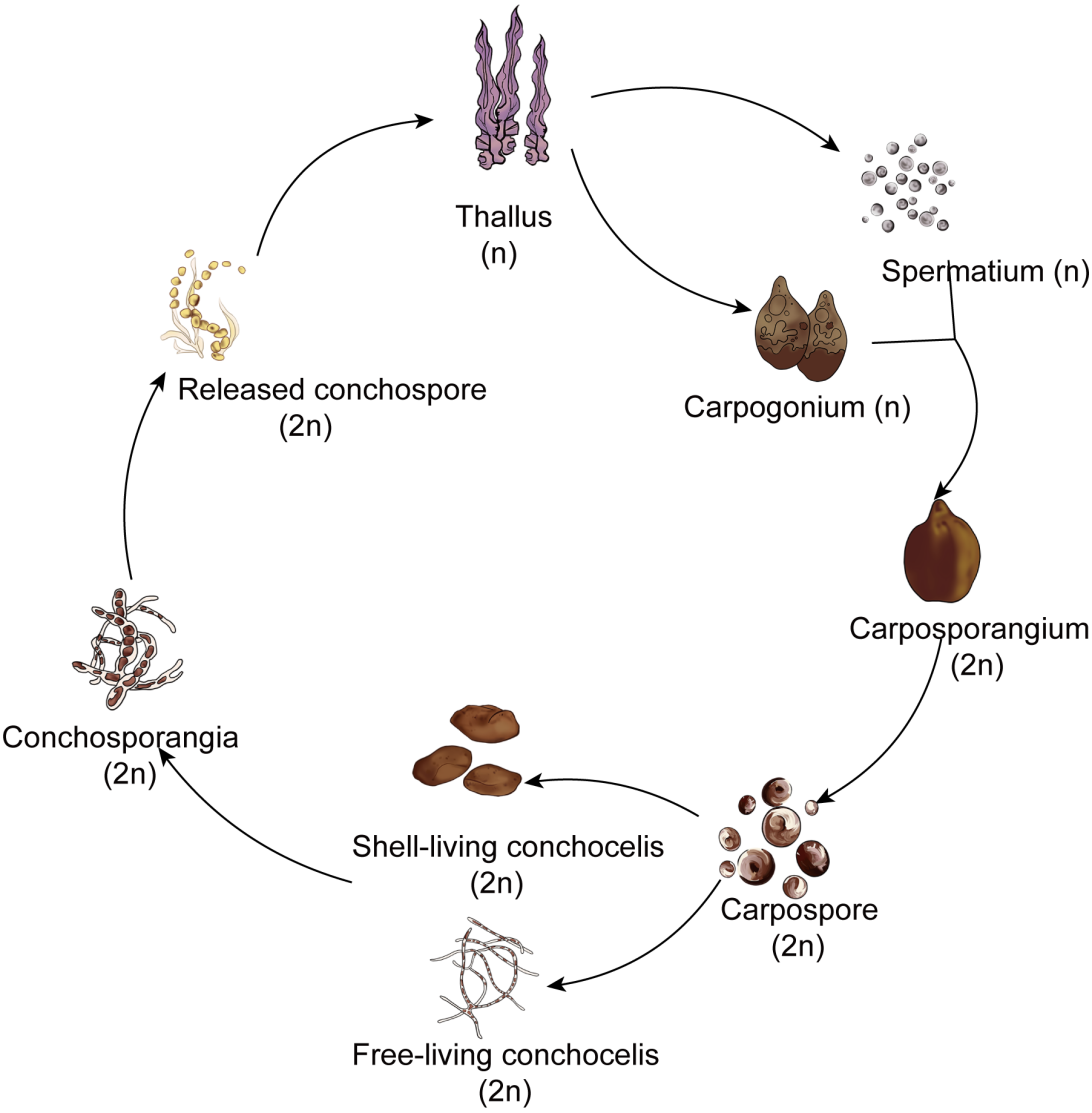
The simplified life history of *Pyropia* and *Porphyra*.

Red algae belong to the Archaeplastida supergroup. Among multicellular red algae, the Bangiales include important marine crops such as *Pyropia* and *Porphyra*. This order represents an ancient lineage with fossil records providing evidence of sexual reproduction dating back 1.2 billion years (Butterfield, 2000). Notably, this is recognized as one of the earliest instances of multicellular eukaryotes engaging in reproduction (Mikami and Takahashi, 2023). These red algae exhibit a heteromorphic haploid–diploid sexual life cycle in which the macroscopic leafy gametophyte (thallus) alternates with the microscopic filamentous sporophyte (conchocelis). The simplified life history of *Pyropia haitanensis* unfolds as follows: Male and female gametes on the thalli fertilize to form diploid carpospores. The released carpospores penetrate the shell, where they undergo development into filamentous conchocelis, residing within the shell. The conchocelis further matures into conchosporangia, eventually releasing conchospores. These spores undergo meiosis, ultimately leading to the development of gametophytic thalli. (**Figure 1**; Lin et al., 2021). Although the prevalent perspective suggests that the large leafy gametophyte is the dominant phase in the life history (Borg et al., 2023), this stage persists for a relatively short time during harsh winter seasons in the rocky intertidal zone. By contrast, the survival of the small conchocelis may be facilitated by its shell-boring habit, which provides a warm aquatic environment that is shielded from excessive light, desiccation, and potential grazers (Varela-Alvarez et al., 2004). Therefore, because the conchocelis persists for a longer duration than the large leafy gametophyte, it seems that this phase should be considered the dominant phase in the life cycle. However, growth within the shell hinders genetic variation in the population and the expansion of the population in geographical locations. Hence, there is a need to detach from the shell and enter the gametophyte generation phase to achieve fertilization and reproduction.

Interestingly, filamentous sporophytes within the shell do not undergo direct meiotic division to form gametophytes. Instead, it takes months for them to produce conchosporangial branches, and only then do mature conchosporangia release conchospores (Lin et al., 2021). Moreover, the conchosporangia do not result from enlargement of the original conchocelis cells; rather, they are newly produced from the conchocelis through tip growth via swelling of the apical cells of the conchocelis branch (Taya et al., 2022). This process may be designed to escape the shell’s confinement, allowing for emergence from the shell and subsequent renewed vegetative growth in preparation for the formation of a large number of gametophytes.

Great differences in cell shape, content quantity, and gene expression profiles are known to exist between conchocelis and conchosporangia (Shen et al., 2000; Varela-Alvarez et al., 2004; Mikami et al., 2019). Mikami et al. (2019) even suggested that the conchosporangia represent a distinct life-cycle generation. These characteristics indicate that the transitions between generations might be regulated in unique ways in Bangiales. In some *Pyropia/Porphyra* species, the transition from conchocelis to conchosporangia is triggered by specific environmental stimuli, such as a shortened photoperiod and temperature upshift (Lin et al., 2021). Under this stimulation, the filamentous cells cease vegetative growth, giving rise to conchosporangial branches. However, it is not clear what triggers this process.

Recent evidence suggests that phytohormones are involved in sexual reproduction of algae. For instance, exogenous methyl jasmonate and polyamines promoted the maturation of cystocarps and the release of spores in *Grateloupia imbricata* and *Hydropuntia cornea* (Guzman-Urióstegui et al., 2012; Garcia-Jimenez et al., 2017). In addition, ethylene has been found to hasten tetrasporangia maturation in *Pterocladiella capillacea* (García-Jiménez and Robaina, 2012), while the ethylene precursor 1-aminocylopropane-1-carboxylic acid (ACC) induced transition from a vegetative to a sexual reproductive phase in *Pyropia yezoensis* and *Pyropia pseudolinearis* (Yanagisawa et al., 2019; Uji et al., 2020). Although few studies have investigated the regulation of conchosporangium development, one study did show that tip growth was accelerated by indoleacetic acid (IAA) treatment in *P. yezoensis* (Taya et al., 2022). The majority of phytohormones are highly integrated with redox or reactive oxygen species (ROS)-mediated signaling (Xia et al., 2015). Owing to its diffusible nature, H_2_O_2_ appears to function as a versatile signaling molecule in red algae (Shim et al., 2022). For example, H_2_O_2_ can prompt vegetative cells to transform into monospores, generating gametophytes asexually in *P. yezoensis* (Takahashi and Mikami, 2017).

Therefore, in this study, we examined changes in *Pyropia haitanensis* phytohormones while focusing on ACC levels to elucidate the mechanisms that regulate the unique transitions that occur in response to environmental fluctuations during conchosporangial development. We found that H_2_O_2_ signaling might serve as the key signal initiating conchosporangia formation and that exogenous ACC substantially enhances H_2_O_2_ production, thereby promoting conchosporangia formation. Moreover, transcriptome profile analysis conducted within one day of exposure to maturation-promoting conditions revealed that the responses of *P. haitanensis* to environmental fluctuations to facilitate conchosporangia development consisted of synthesizing substances, ensuring an adequate energy supply, and regulating the oxidative–antioxidative balance. Exogenous ACC was found to enhance this process.

## Materials and methods

### *P. haitanensis* ZD-1 conchocelis culture and treatment

The free-living conchocelis of *P. haitanensis* (ZD-1 strain, purified progeny of doubled haploid lines) were supplied by the Key Laboratory of Marine Biotechnology in Zhejiang province, China. The conchocelis were cultured in sterilized natural seawater containing nutrients (990 nmol/L KNO_3_, 9 nmol/L FeSO_4_·7H_2_O, 57 nmol/L K_2_HPO_4_, 1 nmol/L MnSO_4_·H_2_O, 54 nmol/L Na_2_EDTA, 0.5 mg/L VB_12_, 5 mg/L VB_1_). This culture was maintained at 20 ± 0.5°C under a light intensity of 30 μmol photons m^–2^ s^–1^ and a 14:10 h (L:D) photoperiod. To induce conchosporangia maturation, the culture temperature was adjusted to 29 ± 0.5°C, the light intensity was reduced to 20 μmol photons m^–2^ s^–1^, and the photoperiod was changed to 8:16 h (L:D). In addition, the concentration of K_2_HPO_4_ in the culture medium was adjusted to 110 nmol/L. The medium was renewed every 7 days during the conchosporangia maturation phase.

The free-living conchocelis of *P. haitanensis* cultured under maturation conditions were treated with either varying concentrations of ACC (0, 1, 5, 10 μM), 5 μM 1-aminocyclobutane-1-carboxylic acid (ACBC), 5 μM cycloleucine, or 5 μM ethephon. Samples in each group were randomly selected at different time points. To assess the impact of H_2_O_2_, conchocelis were subjected to treatments with 5 μM ACC, 5 μM ACC + 0.1 μM diphenylene iodonium (DPI), 5 μM ACC + 0.1 μM N, N’-dimethylthiourea (DMTU), 0.1 μM H_2_O_2_, or 0.1 μM methylviologen (MV). For the group treated with 5 μM ACC, H_2_O_2_ levels and the activity of related enzymes were measured at different time points. In addition, soluble sugar and protein contents were assessed weekly. Simultaneously, samples were collected at 5, 11, and 24 h under mature conditions for transcriptome analysis. After 4 weeks of maturation, the fresh weight was recorded and the relative mass growth rate was calculated using the following formula:


Relative mass growth rate(%/day)=[Ln(Wt/W0)/28]×100%

where W0 is the initial fresh weight (g) and Wt is the fresh weight at 4 weeks (g).

The conchocelis and conchosporangia were also assessed microscopically (Nikon Eclipse 80i, Japan). Specifically, 15 fields were randomly selected to capture images, from which the percentage of conchosporangia relative to the total conchocelis at various time points was determined based on the discernible morphological disparities between them (Shen et al., 2000; Varela-Alvarez et al., 2004) using following the formula:


Percentage conchosporangia (%)=number of conchosporangia/total conchocelis×100%

### The preparation and culture of shell-living conchocelis

Pieces of scallop shells, approximately 1–1.5 cm^2^ in size, were inoculated with conchocelis fragments (broken into lengths of 0.2–0.3 mm) at a density of 100 mg/m². The culture medium was the same as described above. After three days in darkness, the shells were transferred to conditions of 20 ± 0.5°C, 20 μmol photons m^–2^s^–1^, and a 14:10 h (L:D) photoperiod. Following a two-week culture period, excess filaments on the shells were removed, the medium was replaced, and the light intensity was increased to 40 μmol photons m^–2^ s^–1^. Upon conchocelis reaching full coverage of the shells, ACC (0, 1, 5, 10 μM) was introduced into the medium, and the culture conditions were adjusted to induce conchosporangia maturation as described above. Every two weeks, the percentage of conchosporangia was calculated based on the evaluation of the conchocelis and conchosporangia (measured in mm^2^) using a Nikon SMZ18 stereo microscope (Nikon Corporation, Tokyo, Japan). Each treatment involved five replicates and five separate shells.


$$\begin{array}{c} \text{Percent conchosporangia}\;\left(\%\right)\\ =\text{conchosporangia area}/\left(\text{conchocelis area}+\text{conchosporangia area}\right)\times 100\% \end{array}$$

### Release of conchospores

Shells treated with 5 μM ACC and untreated shells exhibiting similar sizes and covered with mature conchosporangia were selected for stimulation of conchospores release (n = 6). Release was implemented in the seawater of the marine area (Meishan bay, Ningbo, China) using operational procedures typically employed during actual *P. haitanensis* aquaculture. Briefly, shells were placed in mesh bags, suspended in seawater overnight, and then returned to the laboratory at 5:00 the next morning. Upon arrival in the laboratory, samples were cleaned with sterile seawater, placed in seawater at 26 ± 1°C, and stirred every hour. The release of conchospores was systematically observed and quantified until the release process concluded.

### Conchocelis viability detection

Free-living conchocelis were treated with different concentrations of ACC for 4 weeks under maturation and non-maturation inducing conditions, after which the viability of conchocelis and conchosporangia was assessed by Evans blue staining. Briefly, samples were suspended in 0.5% (w/v) Evans blue solution prepared in medium, then incubated in the dark at 20°C for 10 min. Next, samples were rinsed with sterile seawater until the blue color disappeared, after which they were placed under a light microscope and 10 randomly selected fields were observed. The Image-J software was then used to calculate the stained area and the total conchocelis area, after which the ratio of these two values was used as the relative survival rate.

### Detection of H_2_O_2_ content and the activity of relative enzymes

Fresh samples collected at various treatment time points were ground under liquid nitrogen and then homogenized in cell lysis buffer (Beyotime Biotechnology, Shanghai) for 10 min to produce a 10% homogenate. Following centrifugation (2,500 × g, 10 min, 4°C), supernatants containing the enzymes and H_2_O_2_ were collected, after which the protein content was determined using the Bradford method. The concentrations of H_2_O_2_ as well as the activities of superoxide dismutase (SOD), catalase (CAT), ascorbate peroxidase (APX), and NADPH oxidase (NOX) were measured using assay kits (Nanjing Jiancheng Bioengineering Institute, Nanjing, China) in accordance with the manufacturer’s instructions.

### Cellular H_2_O_2_ detection

Conchocelis samples cultured under maturation conditions for 4 weeks were extracted from the medium and immersed in 3,3’-diaminobenzidine (DAB) staining solution (1 mg/mL in seawater) for one day at 10°C in the dark. Subsequently, the samples were rinsed twice with 95% ethanol, immersed in absolute ethanol, and heated in a boiling water bath for 10 min. The samples were then transferred onto a slide with 60% glycerol, after which the presence of H_2_O_2_ was visualized under a microscope (Nikon Eclipse 80i, Japan) as a reddish-brown stain formed by the reaction of DAB with endogenous H_2_O_2_.

In addition, the OxiVision™ Blue peroxide sensor in the Amplite Fluorimetric Hydrogen Peroxide Assay Kit (AAT Bioquest, Inc., CA) was used to observe H_2_O_2_ production according to the manufacturer’s instructions. The probe reacts with H_2_O_2_, generating blue fluorescence (Ex = 405 nm, Em = 450 nm).

### Total soluble sugar measurement

Fresh samples weighing 50 mg were finely ground under liquid nitrogen and then extracted with 1 mL of 80% hot ethanol three times for 15 min each. The resulting mixture was then centrifuged at 2,500 × g to isolate the supernatant. To determine the total soluble sugar content, 40 μL of the supernatant was combined with 20 μL of 5% phenol solution and 100 μL of sulfuric acid, after which the absorbance was measured at 492 nm.

### Analysis of phytohormones by LC−MS

A total of 100 mg of lyophilized samples was ground to powder under liquid nitrogen, then subjected to extraction by ultrasonication in 1 mL of 90% MeOH for 15 min followed by an additional overnight incubation at 4°C. The supernatant was then obtained by centrifugation under 3,000 g. Further purification was accomplished through a series of solid-phase extraction column separations using the method outlined by Xin et al. (2020). Eluents obtained from columns (Waters, Milford, MA, USA) were then collected and dried under nitrogen. Next, the dried samples were redissolved in 250 μL of 40% MeOH for subsequent analysis by UPLC-MS using a Thermo Fisher U3000 High Performance Liquid Chromatography system. The analysis employed an ACQUITY UPLC BEH C18 column (100 mm × 2.1 mm) maintained at 30°C. The eluent for separation consisted of 0.05% acetic acid (phase A) and acetonitrile (phase B) applied at a flow rate of 0.4 mL/min under the following gradient program: 0–5 min, 5% B; 5–6.5 min, 85%–100% B; 7–8 min, 100% B; 8–10 min, 5% B.

Mass spectrometry was performed using a Thermo ScientificTM Q Exactive hybrid quadrupole-Orbitrap mass spectrometer equipped with a HESI-II probe. The instrument operated on a Target SIM method in positive mode and negative mode. The capillary voltage was set at 3.5 kV and the capillary temperature was 350°C. The sheath gas was 35 arb and the aux gas was 10 arb. The commercial standards of phytohormones were purchased from Sigma-Aldrich (St. Louis, MO, USA) to establish the quantitative standard curves. The quantities of hormones in the samples were determined by comparing the retention time and MS information with standards. The Thermo Scientific™ quantification software Xcalibur™ 4.0 was used for data analysis. All experiments were performed in triplicate.

### RNA isolation, transcriptome sequencing, and analysis

Free-living conchocelis were divided into seven groups. Among these, those cultured under standard conditions were designated as the control group. In addition, three groups were cultured for 5, 11, and 24 h (M-5h, M-11h, M-24h) under conditions that promoted maturation, while the remaining three groups simultaneously underwent treatment with 5 μM ACC for 5, 11, and 24 h (ACC-5h, ACC-11h, ACC-24h) under identical maturation-promoting conditions.

Samples (100 mg) of each group were ground in liquid nitrogen, after which total RNA extraction was conducted using a Plant RNA Extraction Kit (Magen, Guangzhou, China). RNA-seq libraries were then prepared using an Illumina mRNA-seq Library Preparation kit (Illumina, San Diego, CA) and the Illumina HiSeq platform. Following the removal of low-quality reads, the resulting clean reads were aligned to the *P. haitanensis* genome using HISAT2. The StringTie program (Pertea et al., 2015) was then employed to predict new transcripts, which were integrated with genome annotations to compile the definitive transcriptome set. The reads were subsequently mapped to the transcriptome dataset using Bowtie2 software and then quantified using RSEM. To explore the correlations between samples, principal component analysis (PCA) was conducted using the prcomp package for R. The number of fragments per kilobase per million bases (FPKM) was utilized to calculate the expression levels of all gene transcripts. Differentially expressed genes (DEGs), which were characterized by |log2(fold change)| > 1 and an FDR significance score (padj)< 0.05, were identified through DEseq2 (v.1.22.2). Subsequently, Kyoto Encyclopedia of Genes and Genomes (KEGG)-based annotation enrichment was performed using hypergeometric tests, with an FDR threshold of< 0.05 for KEGG pathways with significant enrichment. For visualization, heatmaps were constructed using Euclidean distances and complete linkage clustering via the pheatmap package for R (v3.3.2, www.r-project.org). The TCseq packages of R were used for fuzzy C means clustering of normalized DEGs. To compare differences among groups, the processed transcriptomic datasets were quantile normalized, transformed, and then subjected to hierarchical clustering. Differentially expressed transcripts within each cluster were further assessed for enrichment of pathways in the KEGG databases. The abundances of pathways with a significance level of *P*< 0.05 were visualized through heatmaps created with TBTools (https://github.com/CJChen/TBtools/releases).

Subsequently, the results were validated through quantitative real-time PCR (qRT-PCR). Amplification reactions were conducted using RNA extracted from the various groups employing a LightCycler^®^96 Real-Time PCR System (Roche, Basel, Switzerland) and the primers shown in **Supplementary Table S1**. The qRT-PCR amplification program comprised the following steps: initial denaturation at 94°C for 30 s followed by 40 cycles of denaturation at 94°C for 5 s, annealing at 60°C for 15 s, and extension at 72°C for 10 s. The relative expression levels were computed using the 2^–ΔΔCt^ method, with *β-actin* serving as the reference gene.

### Statistical analysis

In each experiment, three replicates were established for each group, and the results were expressed as the mean ± standard deviation (SD). Data obtained were analyzed using SPSS version 22.0 (SPSS, Inc., Chicago, IL, USA). Significant differences among various treatment groups were assessed through one-way ANOVA analysis, after which multiple comparisons were conducted using Tukey’s HSD test. *P<* 0.05 was considered to indicate statistical significance.

## Results

### ACC potentially plays a role in the formation of conchosporangia

**Figure 2:**
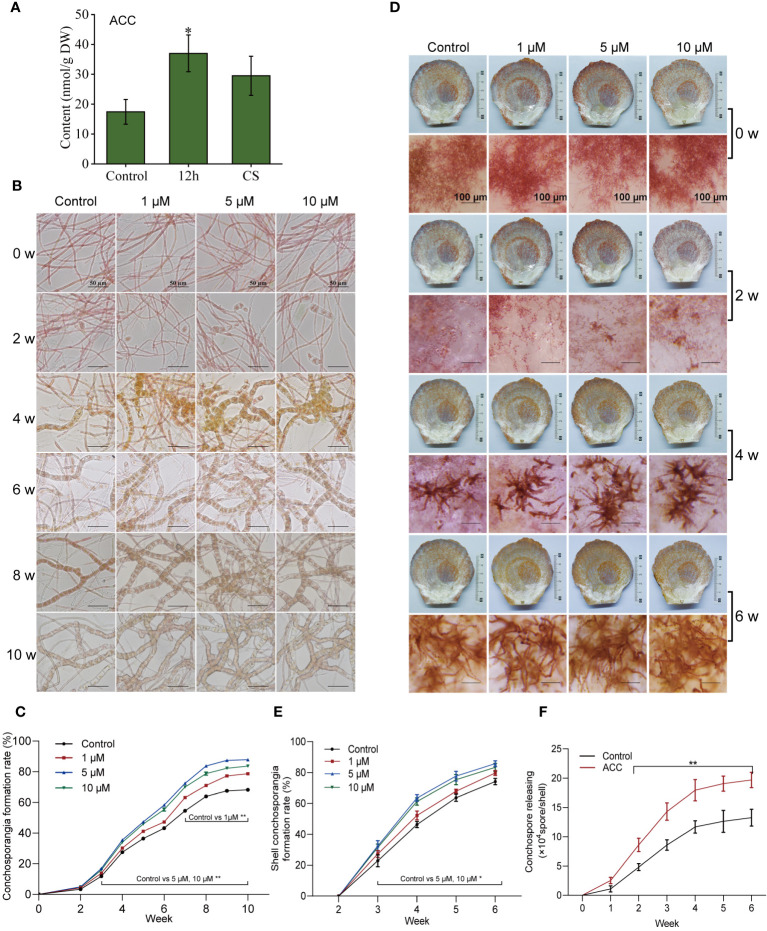
The influence of 1-aminocyclopropane-1-carboxylic acid (ACC) on the development of conchosporangia in *Pyropia haitanensis*. **(A)** ACC levels at various stages of conchocelis exposed to maturation-promoting conditions for 12 hours (12 h) and mature conchosporangia (CS). **(B, C)** ACC’s impact on the formation of free-living conchosporangia at different developmental stages visualized by microscope, with a scale bar of 50 μm. **(D, E)** The effects of ACC on the formation of shell-living conchosporangia at various stages, with a scale bar of 100 μm for filaments emerging from the shells. **(F)** The influence of 5 μM ACC on the release of conchospores. Statistical significance was calculated by one-way ANOVA. ^*^ *P*<0.05 and ^**^ *P*<0.01, compared to the control group (n = 3).

We initially examined the phytohormone levels in conchocelis under three conditions: not subjected to maturation-inducing conditions, exposed to maturation-inducing conditions for 12 h, and mature conchosporangia. A total of nine phytohormones were identified, abscisic acid (ABA), ACC, isopentenyadenine riboside, IAA, isopentenyl adenosine (IPA), jasmonic acid (JA), salicylic acid (SA), trans-zeatin riboside, and zeatin (**Supplementary Figure S1**; **Figure 2A**). Notably, ACC exhibited the highest levels in the conchocelis, reaching 17.43 ± 4.14 nmol/g DW. After adjustment to maturation-inducing conditions for 12 h, only ACC and SA increased significantly. Specifically, the ACC content increased by 1.88 times (*P*<0.05), and salicylic acid content increased by 2.18 times (*P*<0.01). Interestingly, the ACC levels remained relatively high (29.51 ± 6.53 nmol/g) after 10 weeks of maturation, while the salicylic acid levels returned to normal.

The elevated levels of ACC observed during the conchosporangia formation process indicated that it may play a regulatory role in this developmental process. Thus, we investigated the transformation of free-living conchocelis and shell-living conchocelis in response to exogenous ACC. First, we assessed the impact of different concentrations of ACC on the viability of conchocelis cultured for 4 weeks under non-maturing and maturing conditions (**Supplementary Figure S2**). We found that ACC had no effect on conchocelis viability without maturation treatment. However, under maturing conditions, low concentrations of ACC (1 μM, 5 μM) exhibited a protective effect on conchocelis, leading to an increased survival rate. For example, the group treated with 5 μM ACC showed an elevated survival rate of 92.27 ± 2.19%. Therefore, we investigated the effects of ACC concentrations ≤10 μM on conchosporangia formation.

As shown in **Figures 2B, C**, conchosporangial branches were discernible after 2 weeks of maturation, after which the proportion of conchosporangia steadily increased with time. Notably, swelled conchosporangia became apparent under the microscope at week 4, and the proportion of conchosporangia in the control group exceeded 60% by week 10. The addition of ACC exerted a distinct promoting effect on conchosporangia formation, particularly in the group treated with 5 μM ACC. At week 10, the conchosporangia rate reached 87.86%, which was 1.29 times higher than that of the control group (*P*<0.01).

When the shells were subjected to maturation-inducing conditions, the maturation rate of conchocelis in shells was faster than that of free-living conchocelis. After 4 weeks, noticeable branches appeared on the surface of the shell, and the branches on the shell surface turned brown by week 6, indicating conchosporangia maturation (**Figures 2D, E**). Similarly, ACC exhibited a significant maturation-promoting effect, with the optimal effect observed in response to 5 μM. The group treated with 5 μM ACC exhibited distinct conchosporangia as early as 2 weeks into maturation (*P*<0.05). By week 4, the conchosporangia rate reached 63.54%, which was 1.37 times higher than that of the control group (*P*<0.05).

The release treatment was applied to mature shell conchosporangia, and the numbers of released conchospores were calculated (**Figure 2F**). Conchospores from shells continued to be released over time, with a noticeable increase observed after 1 h; however, the release rate began to stabilize after 5 h. ACC treatment significantly enhanced the release of conchospores, with the number of conchospores released in the ACC group reaching 2.0 × 10^5^, or 1.5 times that of the control group, by hour 6 (*P*<0.05).

**Figure 3:**
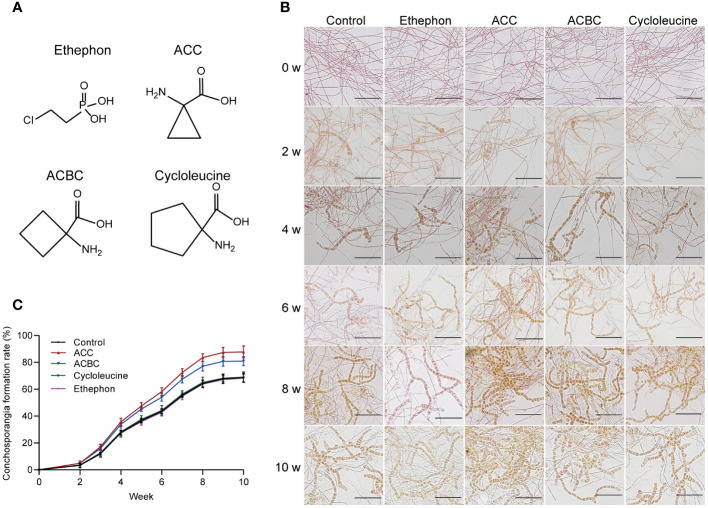
The impact of ACC and its analogs on conchosporangia development. **(A)** Chemical structures of ethephon, ACC and its analogs. **(B, C)** The impact of 5 μM ethephon, ACC and its analog on the formation of free-living conchosporangia at different developmental stages visualized by microscope, with a scale bar of 50 μm.

Next, we investigated whether various ACC analogs could also stimulate conchosporangia formation. All conchocelis were cultured for 10 weeks in the presence of ethephon, ACC, ACBC, and cycloleucine under maturing conditions. The results revealed that ACBC also promoted conchosporangia formation (*P*<0.05), albeit with slightly lower efficacy than ACC. By contrast, cycloleucine and ethephon did not show any promotion of conchosporangia formation (**Figure 3**). These findings implied that ACC can exert its phytohormonal effects independently of ethylene.

### Conchosporangia formation is associated with the H_2_O_2_ signal and ACC amplified this signaling mechanism

**Figure 4:**
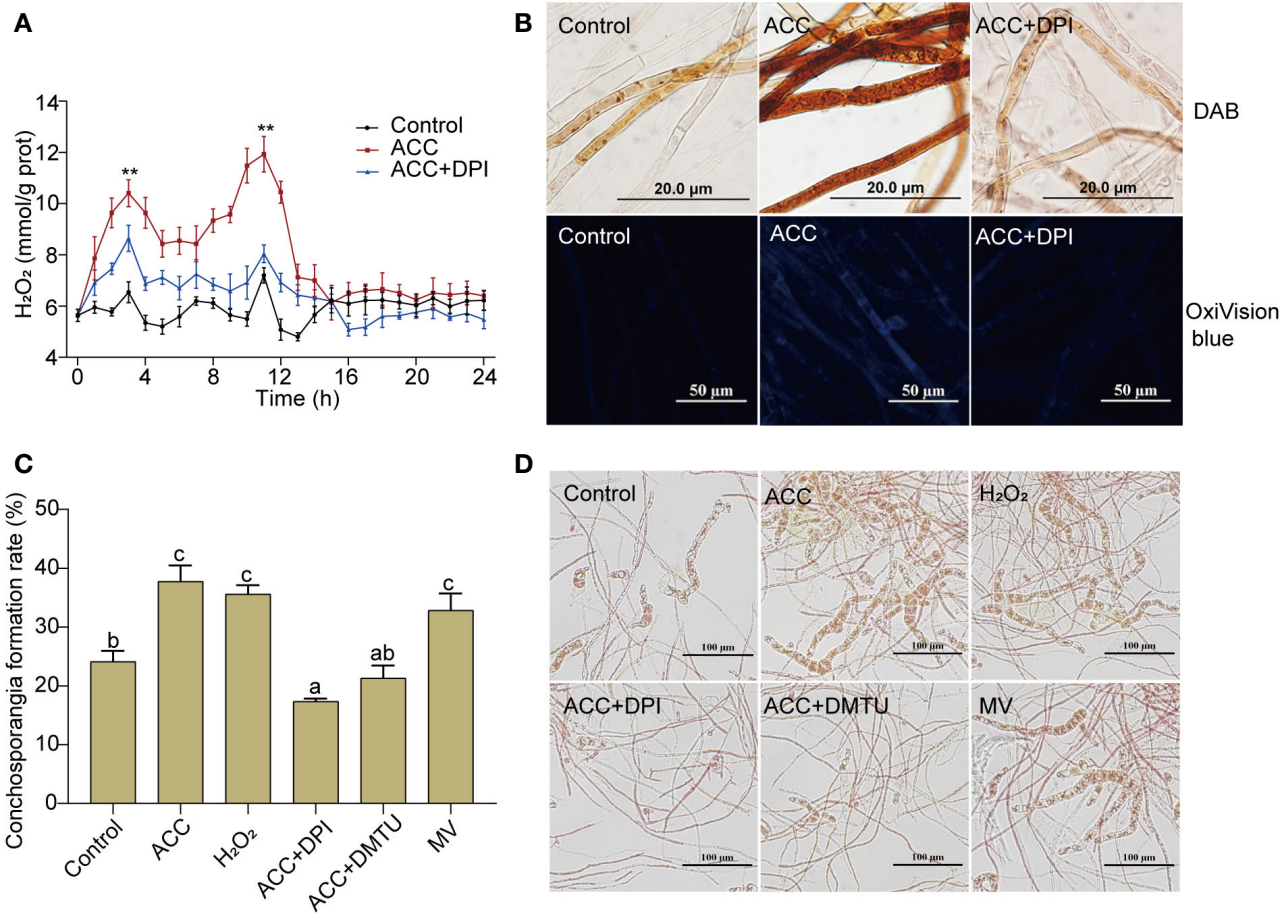
H_2_O_2_ burst in conchocelis exposing to maturation conditions and the promotional effect of ACC. **(A)** H_2_O_2_ generation curve. ^**^ *P*<0.01, compared to the control group (n = 3). **(B)** Micrographs of conchocelis stained with DAB and OxiVision Blue. Scale bars: 20 and 50 μm. **(C, D)** The influence of H_2_O_2_ and its synthetic interfering agents on conchosporangia formation under maturation conditions after 4 weeks. Scale bar: 100 μm. Conchocelis were exposed to maturation conditions and treated with 5 μM ACC, along with the NADPH oxidase inhibitor DPI, the H_2_O_2_ scavenger DMTU, or the superoxide anion generator MV. H_2_O_2_ levels were measured at different time points. At 11 h, the conchocelis were subjected to DAB and OxiVision Blue staining for visualization under a microscope, and at 4 weeks, the conchosporangia formation were observed. Statistical significance was checked by one-way ANOVA, followed by Tukey’s HSD test. Different lowercase letters indicate significant difference among groups at 0.05 level (n = 3).

Although the formation of conchosporangia can be a lengthy process, the triggering events may occur within a short period. We found that when the temperature, phosphorus concentration, and photoperiod changed, a rapid burst of H_2_O_2_ occurred. Notably, there were two distinct bursts that peaked at 3 and 11 h, followed by a return to the initial level (**Figure 4A**). The addition of 5 μM ACC greatly augmented this outbreak. Although the peak times for H_2_O_2_ production remained at 3 and 11 h, the amount produced increased significantly (*P*<0.01). At 11 h, the peak reached 11.93 mmol/g prot, which was 1.66-fold greater than that of the control group at the same time point (*P*<0.01). Simultaneously treating conchocelis with the NADPH oxidase inhibitor (DPI) substantially suppressed ACC-induced H_2_O_2_ production, with the second peak being reduced to close to the level of the control group. To further assess H_2_O_2_ production after inducing maturation for 11 h, we employed DAB, which forms a brown precipitate upon oxidation by H_2_O_2_. As shown in **Figure 4B**, the addition of 5 μM ACC resulted in the conchocelis displaying a dark brown coloration, indicating a substantial generation of H_2_O_2_. Conversely, conchocelis treated with DPI showed significantly reduced staining, confirming the inhibition of H_2_O_2_ generation. Furthermore, the more sensitive H_2_O_2_-specific fluorescent probe OxiVision blue revealed significant blue fluorescence in the ACC-treated group at 11 h under maturation-inducing conditions. However, after DPI treatment, the blue fluorescence was too weak to be captured by the microscope, aligning with the results obtained using DAB.

To investigate the correlation between H_2_O_2_ production and conchocelis maturation, we directly exposed conchocelis to 100 nM H_2_O_2_. Remarkably, our observations revealed that H_2_O_2_ facilitated the formation of conchosporangia (**Figures 4C, D**, *P*<0.01). In addition, while subjecting conchocelis to 5 μM ACC, we concurrently introduced DPI or DMTU (an H_2_O_2_ scavenger) and observed a pronounced inhibition of ACC promotion of conchosporangia formation. By week 4, the formation rate of conchosporangia had decreased to below the levels observed in the control group. Furthermore, treating conchocelis with MV (a superoxide anion generator) alone elevated the conchosporangia formation rate with a promotion effect comparable to that of ACC.

### The transcriptome profile provides potential mechanisms for short-term triggering of the maturation process

Due to the significant generation of H_2_O_2_ at 11 h of maturation, we focused on transcriptional changes in the conchocelis that occurred within 24 h of exposure to maturation conditions. A total of 2.6 × 10^7^ clean reads averaging 150 bp in length were obtained. Of these, 81.60% could be aligned to the reference genome of *P. haitanensis* of 11,321 genes. We noticed a rapid increase in expressed genes in conchocelis over 24 h of promotion treatment. Specifically, the number of genes expressed (FPKM > 0) was 9,958 at 24 h, and the expression of genes increased from 82.62% in the control group to 87.96% (**Supplementary Table S2**). We randomly selected 10 unigenes to validate the RNA-seq analysis using qRT-PCR and found that the expression of these genes in the transcriptome data was generally consistent with the qRT-PCR results (**Supplementary Figure S3**).

**Figure 5:**
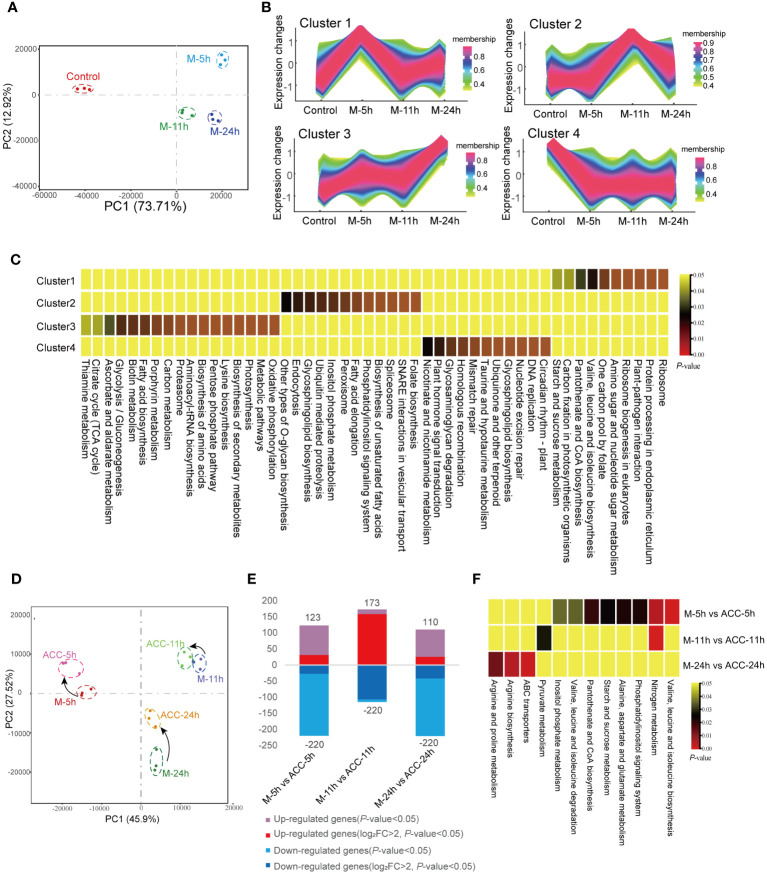
Transcriptional responses of conchocelis to maturation condition stimulation. **(A)** Principal components analysis (PCA) of the transcriptomic profile of individual traits in conchocelis under maturation conditions for 5, 11 and 24 h. **(B)** Clusters of scaled transcript profiles in conchocelis following maturation conditions treatment. Cluster memberships were determined by the similarities of objects measured with respect to subspaces. **(C)** The corresponding heatmap describes significantly enriched KEGG pathways for each cluster. **(D)** PCA of the transcriptomic profile of individual traits in conchocelis in response to ACC treatment. **(E)** Number of differentially expressed genes (DEGs) between different groups. **(F)** Heatmap of significantly enriched pathways for DEGs. M-5h, M-11h, M-24h and ACC-5h, ACC-11h, ACC-24h respectively denote the conchocelis sampled at 5, 11, and 24 h under mature condition stimulation, and coupled with ACC treatment.

The PCA results of the transcriptomes of the control group and the groups induced under maturation conditions for 5, 11, and 24 h were analyzed (**Figure 5A**). A noticeable separation was observed between the control group and the maturation groups, indicating differences in gene transcription. Separations were also observed between the M-5h, M-11h, and M-24h groups, suggesting transcriptional differences, although the relative distances among these groups remained relatively close. After altering the culture conditions for 5 h, the number of DEGs reached 3,434 (control vs M-5h). Subsequently, at 11 and 24 h, the number of DEGs decreased significantly (**Supplementary Figure S4A**), indicating that the DEGs induced by maturation conditions were primarily concentrated in the early stage, specifically the M-5h stage. The Venn diagram shown in **Supplementary Figure S4B** illustrates that 962 DEGs were commonly present in the three groups representing genes that exhibited sustained expression within 24 h of entering the maturation conditions. Subsequent enrichment analysis of these DEGs by KEGG (**Supplementary Table S3**) revealed their involvement in pathways such as ABC transporters, carotenoid biosynthesis, the TCA cycle, glycolysis, nitrogen metabolism, ribosome, and endoplasmic reticulum synthesis. In addition, 1,129 DEGs only showed rapid changes at 5 h. These genes were associated with endoplasmic reticulum protein processing, aminoacyl-tRNA synthesis, and ribosome synthesis. The 10 most enriched KEGG pathways between the control group and the maturation groups were also screened (**Supplementary Figure S4C**). In the M-5h group, the major enriched pathways included those associated with the TCA cycle, carbon fixation, plant–pathogen interactions, and ribosomes. In the M-11h group, the primary pathways were involved in carotenoid biosynthesis, carbon fixation, and ribosomes. For the M-24h group, the predominant enriched pathways were involved in carbon fixation and photosynthesis, etc.

Next, all DEGs were classified into four clusters based on their changing trends (**Figures 5B, C**). KEGG enrichment analysis revealed that genes in Cluster 1 were rapidly upregulated after 5 h of exposure to maturation conditions, after which they declined. These genes were associated with pathways related to protein synthesis and processing, such as ribosome and protein processing in endoplasmic reticulum, etc., as well as pathways involved in carbon fixation and plant–pathogen interactions (*P*<0.05). Cluster 3, which showed consistent upregulation throughout the 24 h maturation process, consisted of pathways associated with oxidative phosphorylation, the citrate cycle, and various amino acid and metabolite synthesis processes (*P*<0.05). Genes in Cluster 4, which were rapidly downregulated, were involved in several pathways related to DNA repair.

Under maturation-inducing conditions, the application of exogenous 5 μM ACC had an impact on the transcriptome profile but did not lead to a large number of DGEs (**Figure 5D**). At 5 h, the number of DEGs between M-5h and ACC-5h was 343 (**Figure 5E**). Similarly, PCA demonstrated that the duration of entry into maturation was the primary factor influencing the differences between groups. After ACC treatment, the principal components of samples from various time points showed closer proximity, indicating a smaller inter-sample difference. Comparison between ACC-5h and M-5h revealed that the upregulated pathways primarily consisted of those involved in nitrogen metabolism, various amino acid metabolism pathways, and starch and sucrose metabolism. However, a comparison of ACC-11h/M-11h and ACC -24h/M-24h revealed fewer enriched pathways, with the main pathways exhibiting upregulation being associated with nitrogen metabolism, pyruvate metabolism, arginine biosynthesis, and ABC transporters (**Figure 5F**).

### Regulating the formation of H_2_O_2_ as a signal-triggering conchosporangia maturation

**Figure 6:**
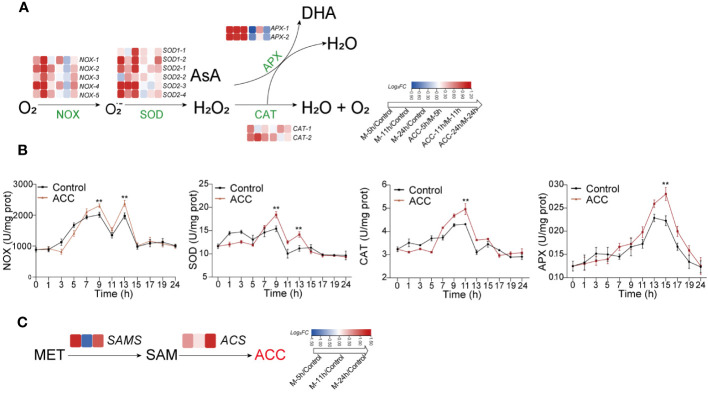
The generation and decomposition of H_2_O_2_. **(A)** Enzyme activity associated with H_2_O_2_ metabolism. **(B)** Heatmap illustrating differentially expressed genes involved in H_2_O_2_ metabolism. **(C)** Heatmap depicting genes related to catalyzing ACC biosynthesis. Conchocelis were subjected to maturation conditions and treated with 5 μM ACC. Enzyme activity was assessed at different time points. Transcriptional profiles of genes were recorded at 5, 11, and 24 hours. The heatmap displays the log2 fold-change values of transcripts. Statistical significance was calculated by one-way ANOVA. ^**^ *P*<0.01, compared to the control group (n = 3).

The above results demonstrated that H_2_O_2_ played a crucial role in regulating the formation of conchosporangia. Therefore, synthesis and metabolism-related genes and the enzyme activities of H_2_O_2_ were investigated. Several NOX- and SOD-encoding genes related to H_2_O_2_ production were identified in the *P. haitanensis* genome, and their expression was generally upregulated within 24 h of applying maturation-inducing conditions (**Figure 6A**). Moreover, enzyme activity increased in response to maturation-inducing conditions, with two peaks being formed. The first peak was observed at 9 h, while the second occurred at 13 h. Genes encoding APX and CAT, which are responsible for metabolizing H_2_O_2_, also showed increased expression in response to changes in culture conditions. The enzyme activities of these enzymes increased, with CAT activity peaking at 11 h and subsequently decreasing and APX activity peaking at 15 h (**Figure 6B**).

The synthesis of ACC occurs through the catalysis of methionine by SAM synthetase (SAMS) to form S-adenosyl-L-methionine (SAM), followed by its conversion under the catalysis of ACC synthase (ACS). We first observed upregulation of the expression of both SAMS and ACS genes after the initiation of maturation (**Figure 6C**), which may explain the increased ACC content. When ACC was added to the culture medium, there was a further increase in *NOX* and *SOD* expression, along with a corresponding elevation in their enzyme activities (**Figure 6B**; *P*<0.01). Upregulation of the APX and CAT genes was not as great, although their enzyme activity levels were still elevated.

### Accumulation of substances is the primary task during conchosporangia formation

**Figure 7:**
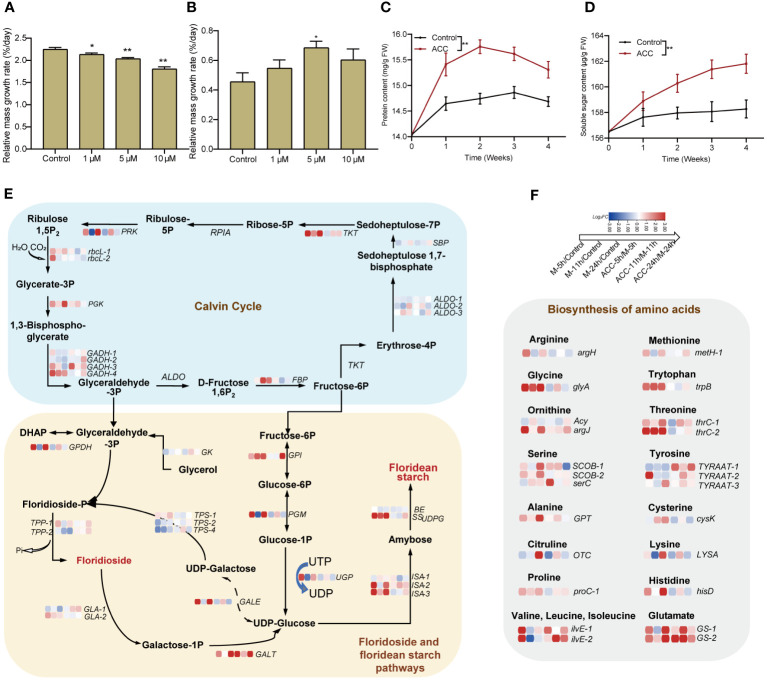
Accumulation of substances in conchocelis under maturation conditions. The influence of ACC on the relative mass growth rate of conchocelis cultivated under **(A)** normal growth conditions and **(B)** maturation conditions for 4 weeks. Statistical significance was calculated by one-way ANOVA. ^*^ *P*<0.05 and ^**^ *P*<0.01, compared to the control group (n = 3). **(C, D)** The influence of 5 μM ACC on protein and soluble sugar contents in conchocelis under maturation conditions. ^**^ *P*<0.01, compared to the control group (n = 3). **(E)** Transcriptional responses of the Calvin cycle and floridoside and floridean starch pathways in conchocelis in response to maturation conditions and 5 μM ACC stimulation. **(F)** Transcriptional changes of genes related to amino acid biosynthesis under maturation conditions and ACC treatment. Heatmap shows the log_2_ fold-change values of transcripts.

In the aforementioned transcriptome analysis, we observed the enrichment of numerous pathways associated with the biosynthesis of various substances, including amino acids, proteins, carbohydrates, and energy metabolism. Consequently, we investigated the growth of free-living conchocelis, which revealed that their daily relative mass growth rate without maturation conditions was 2.25 ± 0.04%. Interestingly, the application of ACC at 10 μM reduced the growth rate to 1.81 ± 0.05%/day (*P*<0.01, **Figure 7A**). Conversely, the daily mass growth rate was lower under maturing conditions than under normal cultivation conditions. The control group exhibited a low growth rate of 0.46± 0.06%/day; however, there was a significant increase following ACC treatment. Specifically, the growth of the 5 μM ACC group surpassed that of the control group by 1.50 times, reaching 0.68 ± 0.04%/day (**Figure 7B**, *P*<0.05). Moreover, the major constituents (soluble sugar and protein) exhibited rapid accumulation after maturation promotion (**Figures 7, D**). Protein levels peaked in the second week, then declined slightly, while sugar content continued to rise. ACC treatment significantly enhanced the accumulation of both protein and sugar (*P*<0.05).

We also analyzed these target pathways in the transcriptome (**Figure 7E**). Within 24 h of exposure to maturing conditions, the Calvin cycle showed an overall upregulation trend. Specifically, genes along the pathway from Rubisoco *rbcL* to the gene catalyzing the formation of fructose-6P (*FBP*) showed significant upregulation (*P*<0.05). In addition, genes involved in the pathway from fructose-6P metabolism to UDP-glucose (*GPI*, *ISA*, and *SS_UDPG_*), which subsequently contribute to floridean starch formation, all showed noticeable upregulation (*P*<0.05). However, genes related to floridioside synthesis were not significantly upregulated, and *TPS2* and *TPS4* were downregulated. Furthermore, genes encoding enzymes catalyzing the conversion of floridoside synthetic precursor UDP-galactose and its breakdown product galactose-1-P into UDP-glucose (*GALE*, *GALT*) were upregulated. Moreover, 18 key genes involved in amino acids synthesis displayed enriched expression, including glycine, tyrosine, and serine exhibiting significant upregulation (*P*<0.01, **Figure 7F**). Following maturation induction, there was an overall upregulation in the expression of ribosomal subunit genes, particularly at 5 and 24 h (**Supplementary Figure S5**). Furthermore, genes related to protein processing and folding in the endoplasmic reticulum membrane and lumen showed upregulated expression. Notably, genes encoding oligosaccharyl transferase (*OST*), which is involved in protein glycosylation, and those encoding the Hsp40 co-chaperone *ERdj3* showed significant upregulation (**Supplementary Figure S6**). Notably, we observed substantial upregulation of multiple heat shock protein genes, including *Hsp40* and *Hsp90*, as well as heat shock transcription factors (*Hsf*) (**Supplementary Figure S7**). The synthesis of a substantial amount of material demands energy, and we found that there were 51 DGEs associated with the tricarboxylic acid (TCA) cycle. Most of these genes exhibited upregulated expression within 24 h during the promotion of maturation, with the majority being upregulated at 24 h (**Supplementary Figure S8**).

The ACC treatment partially enhanced pathways associated with the Calvin cycle, floridean starch pathway, and amino acid biosynthesis. Specifically, genes related to heat shock proteins, such as *Hsf*, showed substantial upregulation after 11 h of treatment (*P*<0.01).

## Discussion

The order Bangiales is a valuable resource for biological studies exploring the evolution of multicellularity, reproductive processes, and responses to environmental fluctuations (Mikami and Takahashi, 2023). Despite this promising aspect, there is currently a lack of physiological and molecular information pertaining to the regulatory mechanisms that control life-cycle transitions in response to environmental changes. The developmental transition from conchocelis to conchosporangia represents a pivotal phase in the life cycle of Bangiales before the transition from the diploid to the haploid generation. It is known that this process is controlled by environmental changes (Waaland et al., 1990), and artificial regulation of this process is important to the success or failure of industrial seedings of *Pyropia* (Lin et al., 2021).

We initially considered the potential alteration of endogenous substances impacted by environmental factors. Phytohormones emerged as the most plausible influencing factors, given their demonstrated role in triggering various growth and developmental processes in higher plants, including cell division, cell enlargement, and cell differentiation (Depuydt et al., 2016). Recent studies have revealed the presence of certain phytohormones in seaweeds akin to terrestrial plants (Mori et al., 2017). Investigations into the effects of phytohormones on seaweed development have highlighted their significance. For example, IAA, JA, and gibberellic acid have been reported to influence the regeneration of *Gelidium floridanum* explants (Batista et al., 2021). Auxin has been shown to regulate tip growth in conchosporangia of *P. yezoensis* (Taya et al., 2022), while cytokinins (zeatin, trans-zeatin riboside, and IPA) may play a role in the regulation of reproductive events in *Ecklonia maxima* (Hofman et al., 1986). In this study, a total of nine phytohormones in *P. haitanensis* were identified by LC-MS analysis. Of note, the content of ACC in the conchocelis was observed to surpass that of other phytohormones, exhibiting a further increase under maturation-promoting conditions. Importantly, the addition of ACC facilitated the formation of conchosporangia, regardless of whether it was applied to free-living or shell-living conchocelis. Additionally, ACC treatment resulted in a significant increase in the release of conchospores after conchosporangia maturation. Collectively, these findings suggest that ACC plays a promotive role in the development and maturation of *P. haitanensis* conchosporangia. However, owing to the diverse action ranges of plant hormones, even those present in small amounts can exert physiological functions (Weyers and Paterson, 2001). Therefore, despite the relatively low content of other hormones in the conchocelis in this study, it does not preclude the possibility that they may play an important role during conchosporogenesis. This aspect will be thoroughly assessed in future studies.

ACC is the direct precursor of the plant hormone ethylene; however, there is increasing evidence that ACC plays a regulatory role in various processes independently from its role in ethylene biosynthesis, including cell cycle and cell division processes, stress responses, stomatal development, and events related to fertilization (Tsuchisaka et al., 2009; Van De Poel and van der Straeten, 2014; Vanderstraeten and van der Straeten, 2017). ACC has also been shown to play various roles in seaweeds. For example, an investigation of *P. yezoensis* indicated that ACC functioned as a signaling molecule influencing sexual reproduction through redox state alterations (Uji et al., 2016, 2020). In addition, a study of *P. pseudolinearis* revealed that ACC had the capacity to modulate the expression of genes involved in the regulation of cell division and cell wall organization, resulting in the formation of spermatangia and parthenosporangia in male and female gametophytes, respectively (Yanagisawa et al., 2019).

ACC contains a three-membered carbocyclic ring. Assuming that the hormone needs to bind with its receptor to transmit signals, structural differences may impact functionality. Our study of several ACC analogs revealed that ACBC, which includes a four-membered carbocyclic ring, also promoted conchosporangia formation, but with slightly lower efficacy than ACC. However, cycloleucine, which contains a cyclopentane ring, did not show similar effects. These findings suggest that the putative ACC receptor structure imposes certain limitations based on the size of the carbocyclic ring and that an excessively large ring may prevent the ligand from entering the binding pocket. This phenomenon is similar to that observed by Uji et al. (2020), who found that treating *P. yezoensis* with ACC and its analogs affected its sexual reproduction. However, additional research is necessary to identify the ACC-binding protein and elucidate the signaling pathway(s) associated with ACC. We also tested ethephon, which can be converted to ethylene and is widely used as a chemical substitute for ethylene treatment in higher plants (Zhang et al., 2010); however, it did not promote conchosporangia formation. Ethylene is synthesized through the conversion of SAM to ACC by the enzyme ACS and then further oxidized to ethylene by ACC oxidases (ACOs) (Yang and Hoffman, 1984). In a previous study, the exogenous application of ethylene was found to promote tetrasporogenesis in the *P. capillacea* (García-Jiménez and Robaina, 2012) and to regulate gene expression during carposporogenesis in *G. imbricata* (Garcia-Jimenez et al., 2018). Ethylene also plays a role in *P. yezoensis* by triggering a transition from a vegetative to a sexual reproductive phase. Even the emission of ethylene has been detected in *P. yezoensis* (Uji et al., 2016). However, in our study, we conducted ethylene detection in the *P. haitanensis* conchocelis using GC-MS, and it was not detected (data not shown). Furthermore, genome sequence analyses have revealed the absence of the ACO gene homolog in red algae (Li et al., 2022), including *P. yezoensis* (Uji et al., 2016) and *P. haitanensis*. Additionally, the red algal genome lacks homologs for receptors and components of the ethylene signaling pathway (Endo et al., 2021). Hence, further investigation is needed to understand the physiological roles that ethylene plays in red algae. Nevertheless, the ACS gene was identified in the genome of red algae. Our data also revealed high expression of the SAMS and ACS genes during conchosporangia development. This not only explains the observed increase in ACC content under maturation conditions but also suggests that ACC itself can act as a signal in red algae, independently of ethylene, during the development of conchosporangia.

There is an intricate interplay between phytohormones and ROS (Raja et al., 2017). For instance, ABA regulates the production of H_2_O_2_ in higher plants, thereby sustaining a positive feedback system for ROS production and stress tolerance. Ethylene has also been identified as playing a role in controlling ROS generation and signaling (Mohanta et al., 2018). In seaweeds, ROS not only are a crucial element in algal immunity (Potin et al., 2002) but also participate in sexual regulation (Takahashi and Mikami, 2017). Among numerous ROS, H_2_O_2_ is considered the predominant ROS involved in cellular signaling responses due to its relative stability (Shim et al., 2022), and its interplay with phytohormones has also been observed in algae. For instance, the application of ACC in *P. yezoensis* increased ROS generation and induced the expression of the *PyRboh*, which encodes NADPH oxidase (Uji et al., 2020). We also detected changes in H_2_O_2_, and found that upon entering maturation conditions, H_2_O_2_ underwent a rapid two-peak burst. Moreover, ACC significantly promoted this burst, after which H_2_O_2_ returned to normal levels and remained unchanged throughout the entire maturing period (10 weeks). It should be noted that this increase could not be ruled out as a response of *P. haitanensis* to heat stress because cultivation conditions involved a substantial increase in temperature from 20°C to 29°C. However, it is very likely that this outbreak was a crucial factor triggering the development of conchosporangia. Therefore, we directly stimulated conchocelis with H_2_O_2_, which confirmed its promotion of the formation of conchosporangia. Cellular H_2_O_2_ is generated by a plasma membrane NOX/SOD system. In this system, NOX transfers electron to molecular O_2_ forming superoxide anion (O_2_˙^−^), followed by dismutation of O_2_˙^−^ by SOD to H_2_O_2_ (Joradol et al., 2019). Here, after treatment with the NOX inhibitor DPI, the observed promotion of H_2_O_2_ production by ACC was inhibited, and this inhibition directly impeded the maturation of conchosporangia. Transcriptome data also revealed upregulated expression of NOX genes in a short period, and enzyme assays showed that NOX enzyme activity peaked at 9 h. We treated the conchocelis with a O_2_˙^−^ generator, MV, which similarly promoted the formation of conchosporangia. Moreover, the enzyme activity and gene expression of SOD were found to be similar to that of NOX, indicating that both NOX and SOD are key proteins involved in the formation of H_2_O_2_. In addition, when the conchocelis was treated with the H_2_O_2_ scavenger DMTU, similar to the effect of DPI, conchosporangia maturation was inhibited. These results suggest that H_2_O_2_ acts as a rapidly responsive signal under promoting conditions, and ACC achieves its role in promoting conchosporangia development by amplifying this signal.

The excessive production of H_2_O_2_ will cause oxidative damage to cells; however, organisms possess the ability to regulate this balance. In a previous study of *P. yezoensis*, gametophytes treated with ACC exhibited overexpression of antioxidant genes (Uji et al., 2016). In our study, we observed that the enzyme activities of two key antioxidative enzymes involved in H_2_O_2_ decomposition, peroxidases (mainly APX) and CAT, increased, but the initiation of activation was slower than that of NOX and SOD. Specifically, they reached peak activities at 11 and 15 h, respectively, indicating that they played a role after the peak production of H_2_O_2_. Although this suggests their involvement in the clearance of excessive H_2_O_2_, their genes were upregulated at the transcriptional level in the early stages of conchosporangia formation. We also observed that ACC played a regulatory role throughout the entire process.

During the development of vegetative conchocelis into conchosporangial branchlets, a series of changes can be observed in cell morphology such as increased cell diameters, enlarged vacuoles, strong pigmentation, and cell walls becoming rough (Shen et al., 2000). Unlike conchocelis filaments that form branches only infrequently, conchosporangia exhibit nutritive proliferation, producing numerous branches (Khoa et al., 2021). This indicates that not only are new cells formed during this process but also there is also an accumulation of cellular contents. Because the carbon fixation rate of conchosporangial branchlets is lower than that of conchocelis, their growth rate was expected to be lower. However, there was still an increase in soluble sugars and proteins during conchosporangia formation, which has previously been reported (Chen et al., 2007); therefore, it is likely to be an energy-consuming process. Mikami et al. (2019) used transcriptome analysis to compare gene expression among three life cycle stages in *P. yezoensis*, thalli, conchosporangia, and conchocelis. They found that the gene expression profile of conchosporangia differed from that of thalli and conchocelis. Specifically, the annotated unigenes were predominantly classified into terms related to “translation, ribosomal structure, and biogenesis” and “cell cycle control, cell division, and chromosome partitioning” in the conchosporangia. In our study, we observed transcriptional changes at 5, 11 (peak H_2_O_2_), and 24 h after promoting maturity, which indicated that gene transcription was highly sensitive to changes in environmental factors. Within 5 h, 3,434 DGEs appeared, and pathways responding rapidly within 5 h included a series of amino acid and protein synthesis pathways, such as those involved in endoplasmic reticulum protein processing, aminoacyl-tRNA synthesis, and ribosome production. While the pathways enriched within 24 h involved the TCA cycle, glycolysis, ribosomes, and endoplasmic reticulum synthesis. In addition, these pathways were upregulated along with those involved in carbon fixation and oxidative phosphorylation. We also conducted a detailed analysis of pathways involved in the Calvin cycle, TCA cycle, electron transport chain, synthesis of multiple amino acids, as well as ribosomal subunit and protein processing and folding. The results revealed rapid responses of these pathways to changes in cultivation conditions. The starch metabolic pathway also indicated that fixed carbon tends to accumulate in floridean starch rather than floridoside, serving as a short-term carbon reservoir (Martinez-Garcia and van der Maarel, 2016). This is consistent with the findings reported by Mikami et al. (2019) and highlights the significant roles that protein and sugar synthesis play in this process, along with the substantial energy demand for synthesis. ACC enhances this process, leading to a significant increase in soluble sugars and proteins, which results in a noticeable increase in the relative mass growth rate. This positive regulation was reflected in the transcriptional upregulation of the mentioned pathways. The accumulation of substances during the conchosporangia stage is likely a preparation for the later release of conchospores and meiotic division.

Lin et al. (2021) also conducted a transcriptome analysis and found that phosphorus metabolic processes, lipid metabolism, and the phosphatidylinositol (PI) signaling system are important metabolic pathways during conchosporangia maturation in *P. haitanensis*. Moreover, they identified a diacylglycerol kinase gene (*PhDGK1*) as a key factor in PI turnover that initiates PI regeneration, suggesting it might be a candidate hub gene for the promotion of the maturation of conchosporangia. We also observed the transcriptional levels of this gene within one day of promotion and found that it is not a rapidly responsive gene. Lin’s results also showed that this gene was only upregulated after 7 or 28 days (in different strains), indicating that it may regulate the later stages of maturation rather than being involved in its initiation.

In a study by Uji et al. (2016), treating *P. yezoensis* with ACC resulted in gametophytes exhibiting overexpression of chaperone-related genes. In the present study, heat stress induced a significant upregulation of Hsp genes, aligning with the protective response of organisms to heat stress. In addition, some Hsp genes were further upregulated after ACC treatment, indicating that ACC plays a protective role in facilitating cell development toward conchosporangia.

## Conclusion

Bangiales exhibit a unique intermediate phase, the conchosporangia stage, during the transition from the diploid sporophyte to the haploid gametophyte. To date, initiation of this transitional stage has not been well understood. In this study, we found that changes in environmental conditions promote the rapid release of H_2_O_2_, which might serve as the key signal initiating conchosporangia formation. Moreover, ACC substantially enhanced H_2_O_2_ production, thereby promoting the formation of conchosporangia. To prevent excessive oxidative damage caused by H_2_O_2_, cells mobilize antioxidant mechanisms and heat shock proteins to regulate their balance. The maturation conditions induced an upregulation of the synthesis of components within 1 day, along with the modulation of energy supply to ensure normal progression. In addition, exogenous ACC was found to enhance the entire process. These findings shed light on the regulation of the sexual life cycle of red algae and provide insight into the functions of phytohormones in these organisms. Moreover, given that the maturation of conchosporangia strongly affects the yield and quality of *P. haitanensis* thalli, the results presented herein will lead to new avenues for industrial seedling regulation.

## Data availability statement

The data presented in the study are deposited in the NCBI SRA database, accession number PRJNA936596.

## Author contributions

TN: Writing – original draft. HC: Conceptualization, Supervision, Writing – review & editing. HQ: Methodology, Writing – original draft. JC: Data curation, Formal analysis, Writing – review & editing. QL: Methodology, Resources, Writing – review & editing. RY: Resources, Writing – review & editing. PZ: Investigation, Resources, Writing – review & editing. TW: Investigation, Writing – review & editing.
