# Supplementary material for: H2O2 drives the transition from conchocelis to conchosporangia in the red alga Pyropia haitanensis with promotion facilitated by 1-Aminocyclopropane-1-carboxylic acid
Source: Front Plant Sci. 2024 Mar 12;15:1379428. doi: 10.3389/fpls.2024.1379428 (PMC10963560; doi:10.3389/fpls.2024.1379428)
Supplement: Supplementary file 2 [file DataSheet_2.docx]

Supplementary Material

## Supplementary Figures


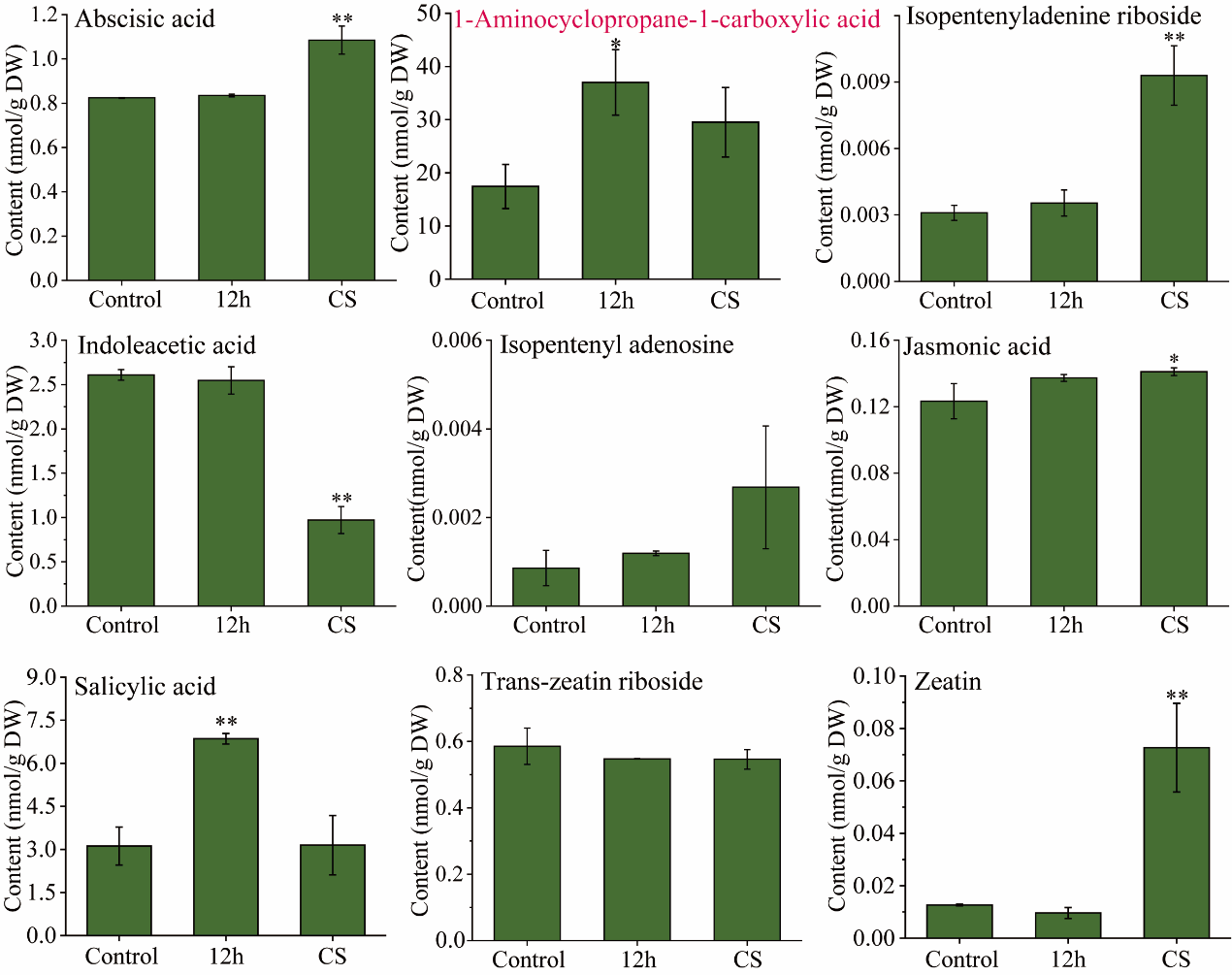


**Supplementary Figure 1.** Phytohormone contents at different stages of conchosporangia development in *Pyropia haitanensis*. Exposing to the maturing-promoting conditions for 12 hours (12 h) and the mature conchosporangia (CS).


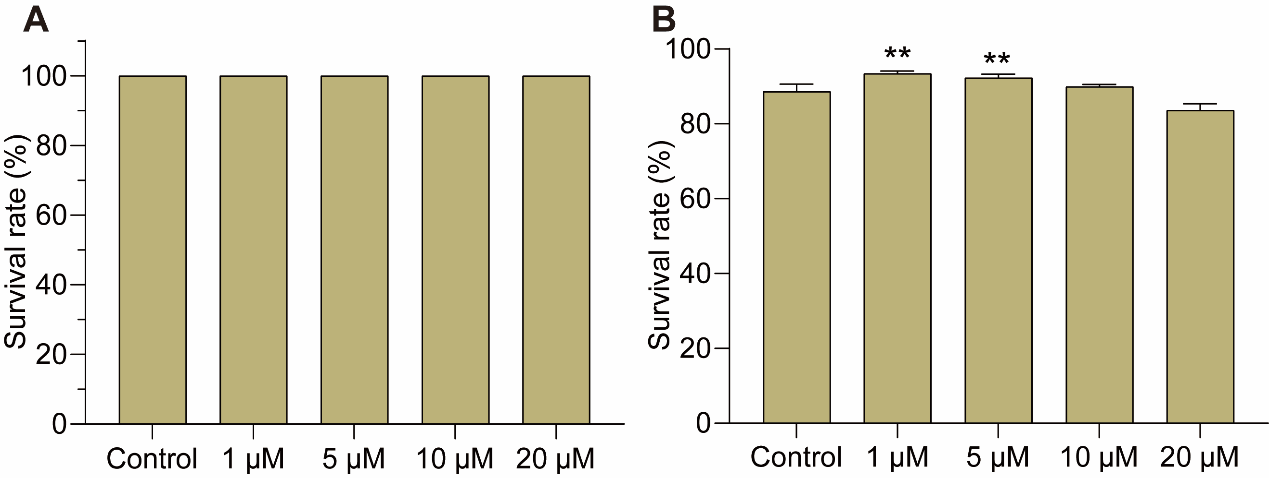


**Supplementary Figure 2.** The impact of ACC on the viability of free-living conchocelis. The survival rate of conchocelis after cultivation under non-maturing (A) and maturing conditions (B) for 4 weeks. **P <* 0.05 and ***P <* 0.01, compared to the control group (*n* = 3).


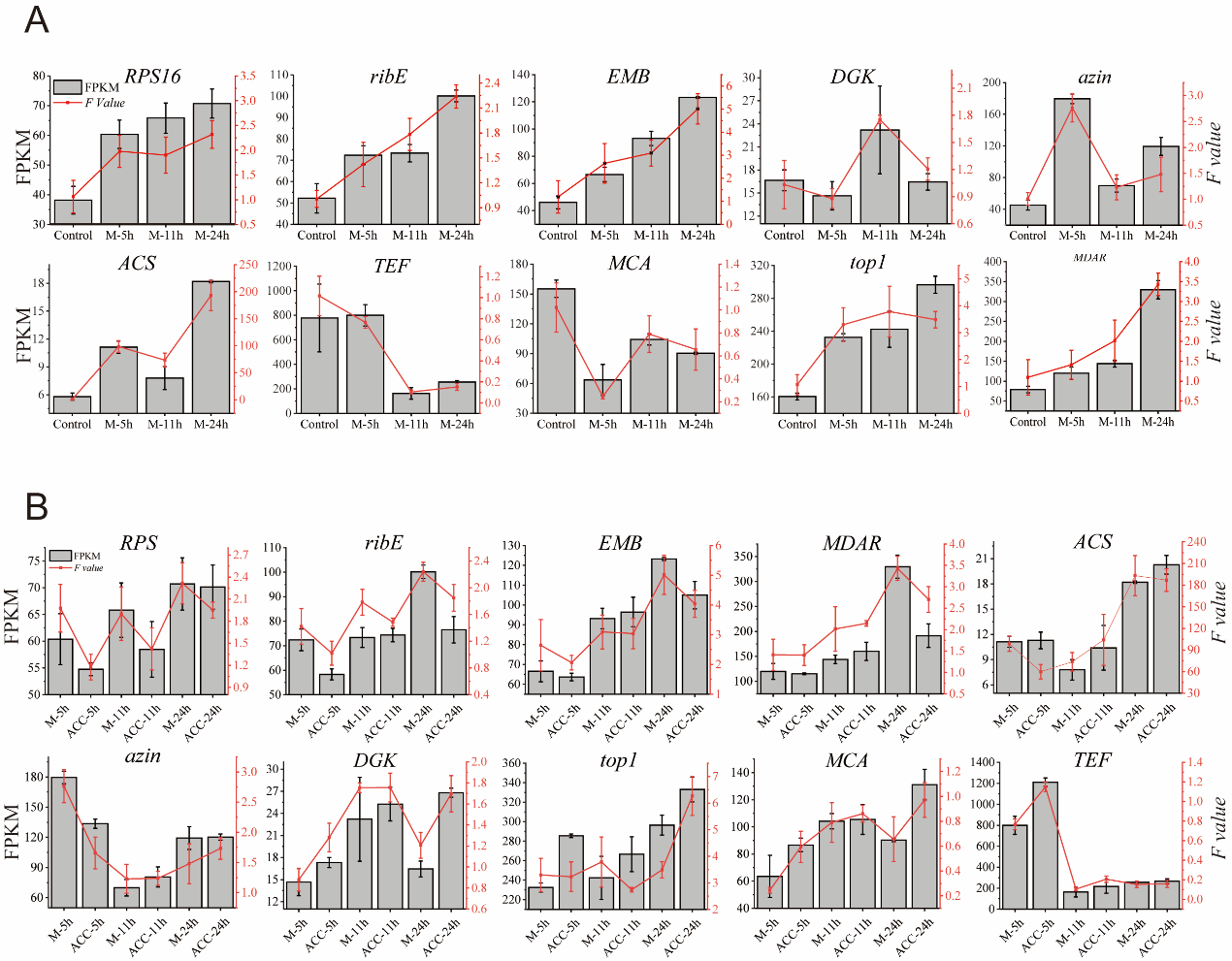


**Supplementary Figure 3.** Validate the expression of randomly selected unigenes by qRT-PCR. (A) The gene expression at various time points within a 24-hour period under maturation conditions. (B) The gene expression of genes at different time points within a 24-hour period under maturation conditions with a 5 μM ACC treatment. Error bars indicate standard errors of the means (n = 3).


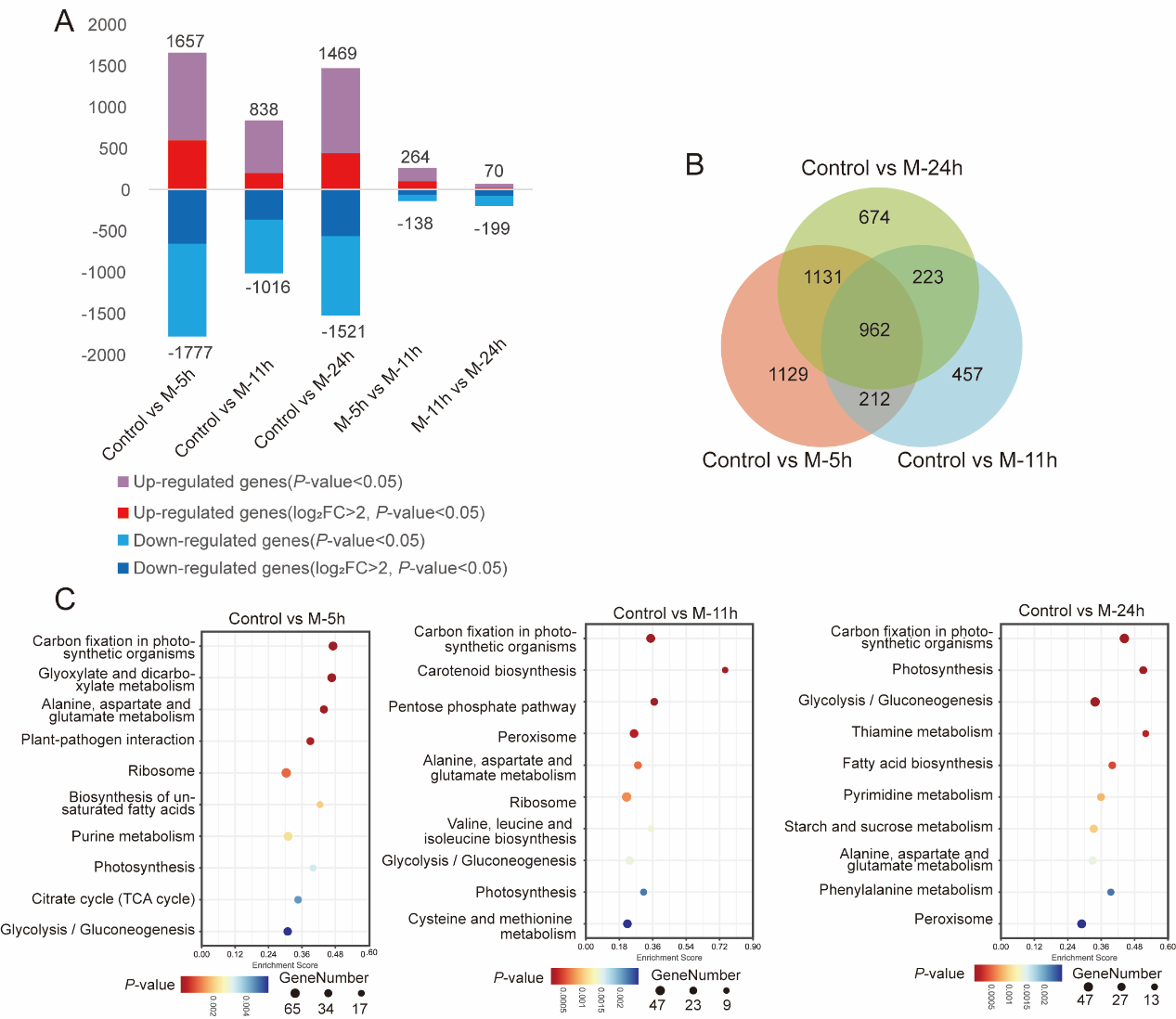


**Supplementary Figure 4.** Transcriptional responses of conchocelis to mature conditions stimulation. (A) Number of differentially expressed genes (DEGs) between different groups. (B) A Venn diagram of DEGs. (C) KEGG enrichment of DEGs between contrasted groups. M-5h, M-11h and M-24 h respectively represent the conchocelis at 5, 11, and 24 hours under mature condition stimulation.


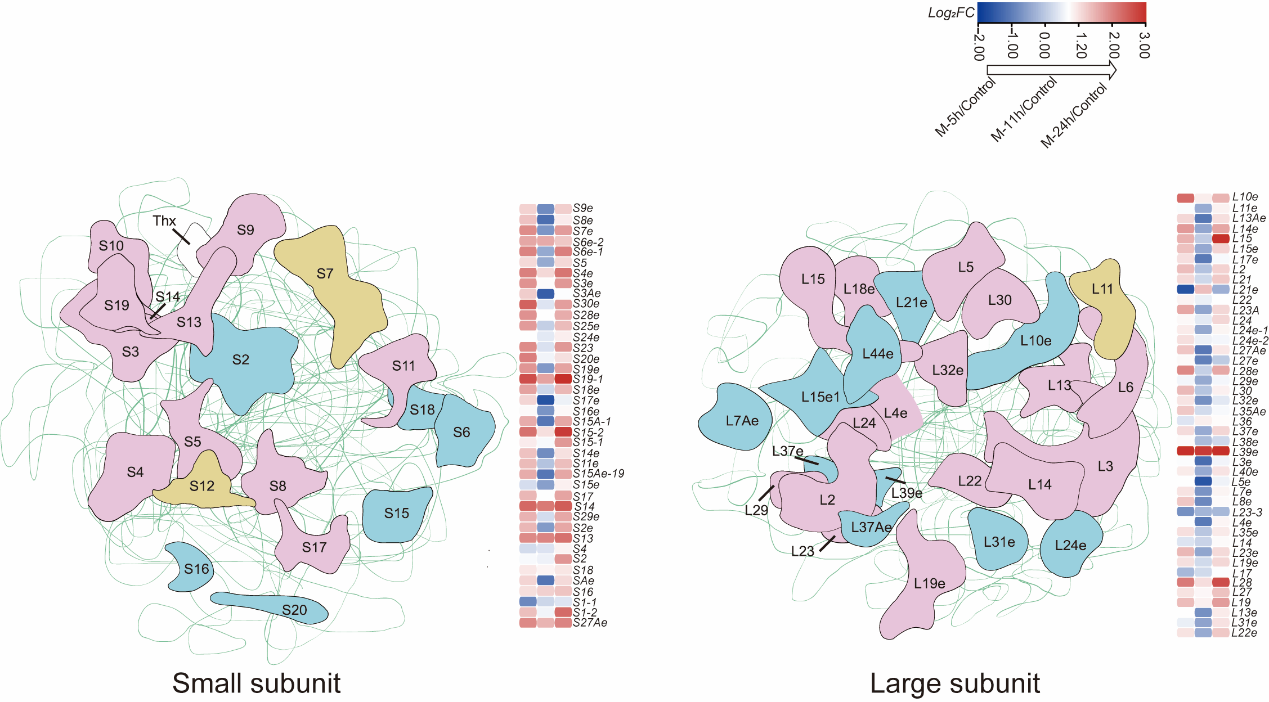


**Supplementary Figure 5.** Expression patterns of ribosomal genes in conchocelis under mature conditions stimulation. Heatmap shows the log2 fold-change values of transcripts.


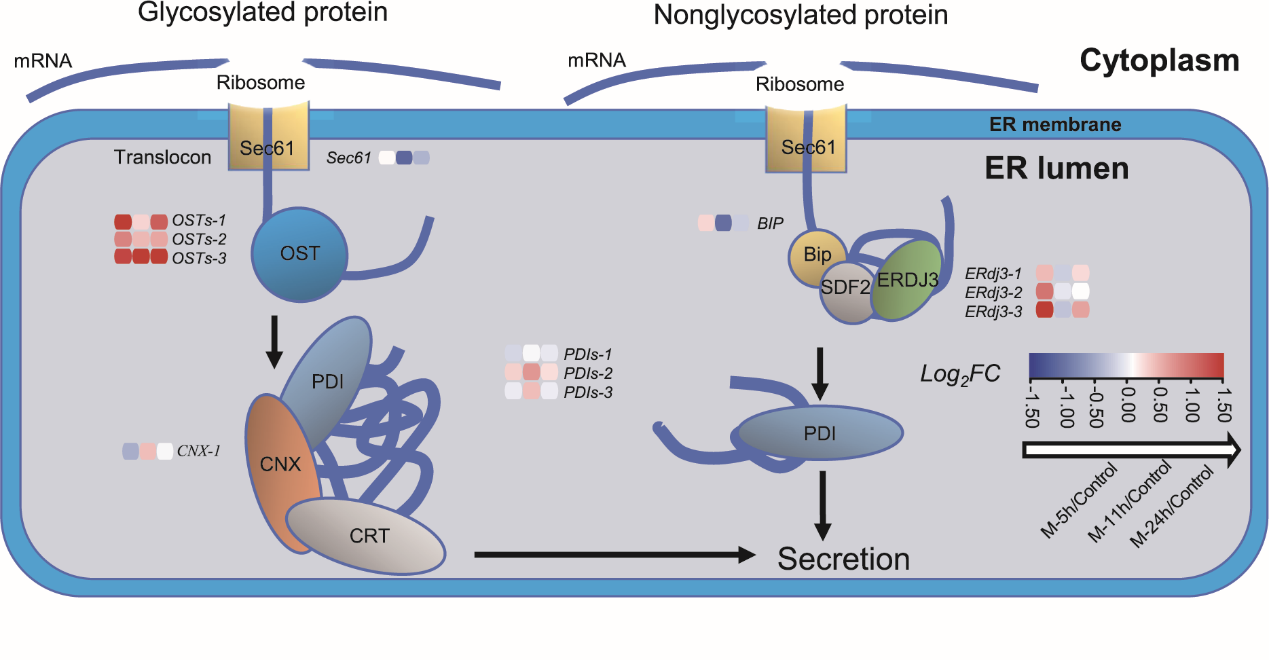


**Supplementary Figure 6.** Expression patterns of endoplasmic reticulum genes in conchocelis under mature conditions stimulation. Heatmap shows the log_2_ fold-change values of transcripts.


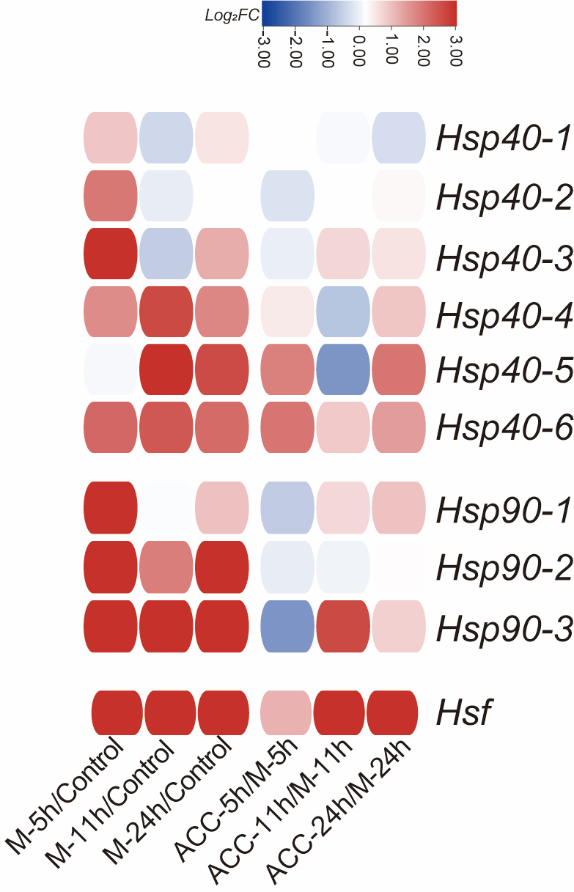


**Supplementary Figure 7.** Expression patterns of heat shock proteins and heat shock transcription factors in conchocelis following treatment with 5 μM ACC under conditions simulating maturation. Heatmap shows the log_2_ fold-change values of transcripts.


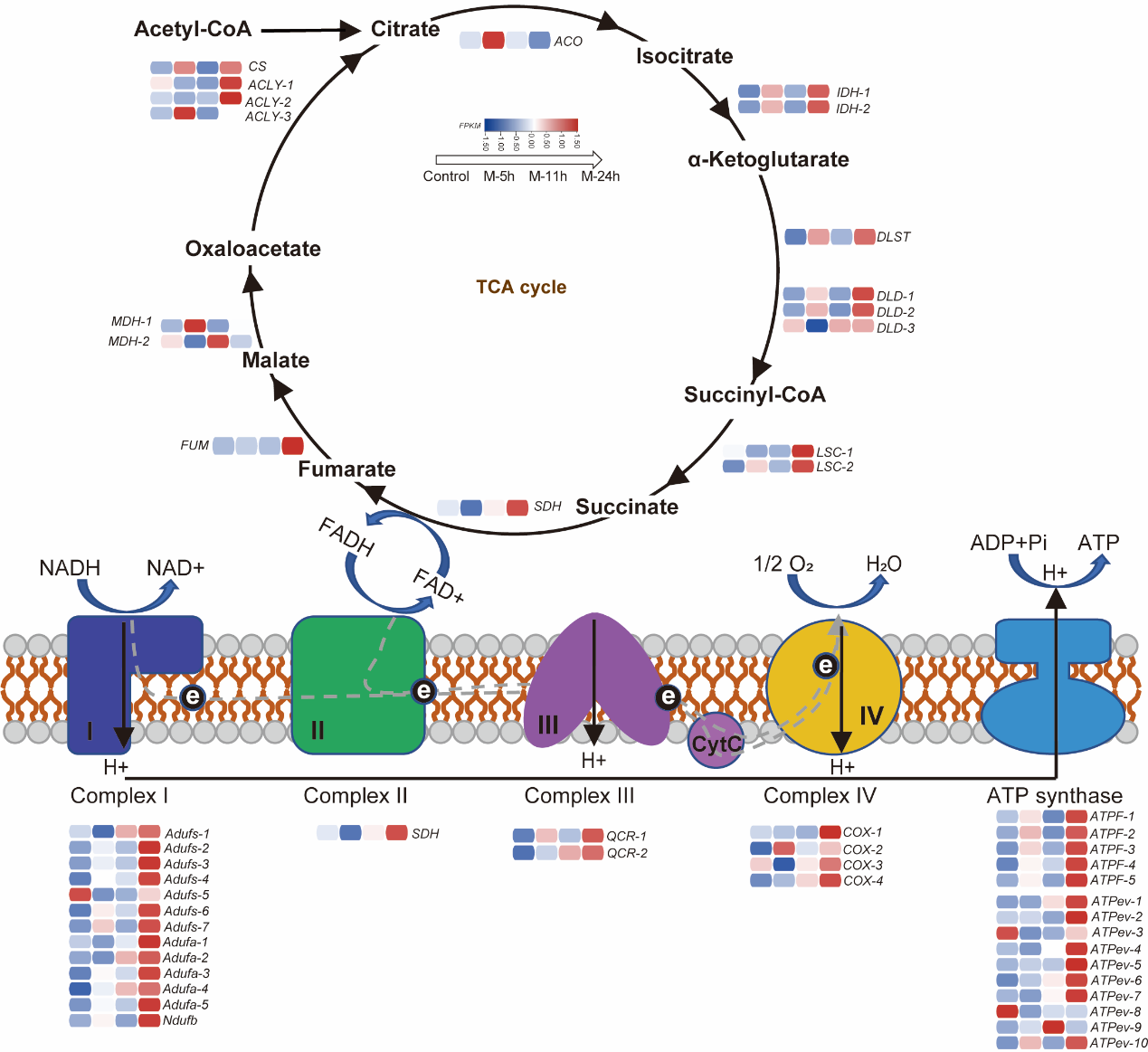


**Supplementary Figure 8.** Transcriptional responses of the tricarboxylic acid (TCA) cycle and electron transport chain systems in *P. haitanensis* conchocelis to mature condition stimulation. The average FPKM values of each gene (n = 3) for different groups are shown in the heatmaps following z-score normalizations.

## Supplementary Tables

**Supplementary Table 1** Primers for qRT-PCR

| **Primers** | **Sequences（5’-3’）** | **PCR products (bp)** |
| --- | --- | --- |
| *Nhβ-actin* | F: GGTGGTGATTGACAATGGGTCT | 193 |
|  | R: CAGCGGGTACTTGATGAGCAG |  |
| *NhTEF* | F: AAGACGCCCAACCAGTACATC | 145 |
|  | R: TCTTCTCCTCATTCTGCCACA |  |
| *NhRPS16* | F: GACGTCGTTGGGAGCTACAAC | 106 |
|  | R: CCGACAGCCAGTACTTGATGC |  |
| *NhribE* | F: GCTCCGCCACAATCTTCTCAA | 116 |
|  | R: TATGATGATCCCCTACACGCA |  |
| *NhEMB* | F: GGCTTCTACTATGACTTTGACGC | 88 |
|  | R: GCTTGATAATCTTGTCCATCTCCT |  |
| *NhMDAR* | F: GTCACCGTTTTCGTCTCCCAC | 112 |
|  | R: GCCACCCAGGATCACATACTT |  |
| *NhACS* | F: TTCTCCAAGGACTTTTGTGGC | 105 |
|  | R: GACGGACGAAAAGTAGGTGAC |  |
| *Nhazin* | F: AGCTACGCAACTGCCCTAGAT | 147 |
|  | R: CCCTACAATCTCATCAAACGTG |  |
| *NhDGK* | F: GACGTCGTTGGGAGCTACAAC | 106 |
|  | R: CCGACAGCCAGTACTTGATGC |  |
| *Nhtop1* | F: GCAAGCAAAACTACCTGGACC | 106 |
|  | R: CAGGAGAACTTGGTCATCAGAG |  |
| *NhMCA* | F: ACGATGAAGTCCTAACCCTGG | 107 |
|  | R: TCTCGTCCAGACCATCCTCTT |  |

**Supplementary Table 2** Transcript gene expression

| **Group** | **Total gene** | **Expressed gene** | **Expression rate** |
| --- | --- | --- | --- |
| Control | 11321 | 9353 | 82.62% |
| M-5 h | 11321 | 9860 | 87.09% |
| M-11 h | 11321 | 9912 | 87.56% |
| M-24 h | 11321 | 9958 | 87.96% |
